# Potential of AKR1B10 as a Biomarker and Therapeutic Target in Type 2 Leprosy Reaction

**DOI:** 10.3389/fmed.2018.00263

**Published:** 2018-09-24

**Authors:** Cleverson T. Soares, Luciana R. V. Fachin, Ana P. F. Trombone, Patricia S. Rosa, Cássio C. Ghidella, Andrea F. F. Belone

**Affiliations:** ^1^Department of Anatomic Pathology, Instituto Lauro de Souza Lima, Bauru, Brazil; ^2^Department of Health Science, Universidade do Sagrado Coração, Bauru, Brazil; ^3^Division of Research and Education, Instituto Lauro de Souza Lima, Bauru, Brazil; ^4^Ambulatory of Leprosy, Jardim Guanabara Health Center, Rondonópolis, Brazil

**Keywords:** leprosy, reaction type 2, AKR1B10, biomarker, therapeutic target

## Abstract

The *AKR1B10* (aldo-keto reductase family 1 member B10) gene has important functions in carcinogen-induced neoplasia. AKR1B10 is also expressed in type 2 reaction leprosy patients (R2). We measured the expression of AKR1B10 in the skin lesions of patients with leprosy by immunohistochemistry from biopsies that encompassed the spectrum of types of leprosy, based on the Ridley and Jopling classification [10 samples each of tuberculoid (TT), borderline tuberculoid (BT), mid-borderline (BB), and borderline lepromatous (BL) lesions; four samples of lepromatous lesions (LL)], reactional leprosy [14 samples of type 1 Reaction (R1) and 10 samples of type 2 Reaction (R2)], and biopsies from 9 healthy control (HC) subjects. In addition, 46 lepromatous lesions (BL and LL), 45 lepromatous lesions in regression, and 115 R2 lesions were included. Eight of 10 R2 samples (80%), 3 of 46 active BL and LL samples (6%), 23 of 45 BL and LL samples in regression (51%), and 107 of 115 R2 samples (93%) were positive for AKR1B10, differing significantly between all groups (*p* < 0.05). AKR1B10 expression was highest in the cytoplasm of macrophages. Thus, AKR1B10 is overexpressed on the lepromatous side (BL and LL) in samples that are in regression, especially type 2 reaction-associated lesions, rendering it a potential marker of type 2 reactional episodes of leprosy and a target of drugs against reactional episodes.

## Introduction

The *AKR1B10* (aldo-keto reductase family 1 member B10) gene encodes a member of the aldo-keto reductase superfamily, which consists of over 40 enzymes and proteins (1, 2). Its ability to reduce several aldehydes and its expression in the luminal cells of the gastrointestinal mucosa suggest that it mediates the detoxification of reactive aldehydes in digested food before nutrients are distributed to other organs (1). AKR1B10 interferes with cell survival by modulating lipid synthesis, mitochondrial function, and oxidative status, implying that it is an important protein in cell survival mechanisms (3). In the intestine, AKR1B10 is expressed specifically by the epithelial cells and is secreted into the lumen (4). It is also commonly expressed in the adrenal gland and might have a significant function in liver carcinogenesis. AKR1B10 is overexpressed in neoplasms of various organs and systems, such as the lung, breast, gastrointestinal tract, pancreas, head, and neck (4, 5).

Overexpression of AKR1B10 is an initial event in the carcinogenesis of lung, liver, pancreatic, and breast cancer (6–10). Based on its overexpression in neoplastic cells, it is considered a tumor marker, and it can be detected *in situ* and in the blood (11–13). Alterations in *AKR1B10* expression have been observed in non-neoplastic diseases, such as atopic dermatitis and diabetes (14, 15). *In vitro* studies have shown that AKR1B10 is present in the lysosomes and is secreted with cathepsin D, a lysosomal marker (16, 17)

Leprosy is a chronic infectious disease that is caused by *Mycobacterium leprae (M. leprae*) (18). It is an important public health issue in Asian, African, and South American countries (18). Because leprosy is a spectral disease that can evolve over many years or decades, its clinicopathological characteristics change slowly but continuously—sometimes with overlapping clinical and histopathological patterns (19, 20).

According to the Ridley and Joplin (R&J) classification system, leprosy is subdivided into polar [tuberculoid (TT) and lepromatous (LL)] and intermediate forms [borderline tuberculoid (BT), mid-borderline (BB), and borderline lepromatous (BL)]; (21). It is believed that this behavior is caused by the low antigenicity of *M. leprae*, which stimulates low-intensity immunocellular reactions. However, episodes of abrupt onset of an inflammatory reaction in cutaneous neural lesions, which are generally more intense and potentially destructive, can be intercalated into the evolution of the disease.

These episodes are called leprosy reactions (19, 20, 22), of which there are 2 types. The type 1 reaction (R1) occurs in patients with varying degrees of preservation of cellular immunity, specific to *M. leprae*. The type 2 reaction (R2), corresponding to erythema nodosum leprosum and its variants develops in patients in whom little cellular immunity is preserved or absent (19, 20, 22). During these episodes, neurological lesions usually worsen with the destruction of the neural branches and tissues, which can cause functional disabilities and permanent sequelae (19, 20, 22).

There are few studies on the association between AKR1B10 and leprosy. Recently, we reported that AKR1B10 was overexpressed in skin biopsies of patients with leprosy, especially those with the type 2 reaction (23). Thus, AKR1B10 has emerged as a potential marker of and therapeutic target in type 2 reactions. The aim of this study was to examine the expression of AKR1B10 in leprosy skin lesions by immunohistochemistry (IHC) and identify the cells in granulomas and skin tissue that express this marker.

## Materials and methods

**Figure 1:**
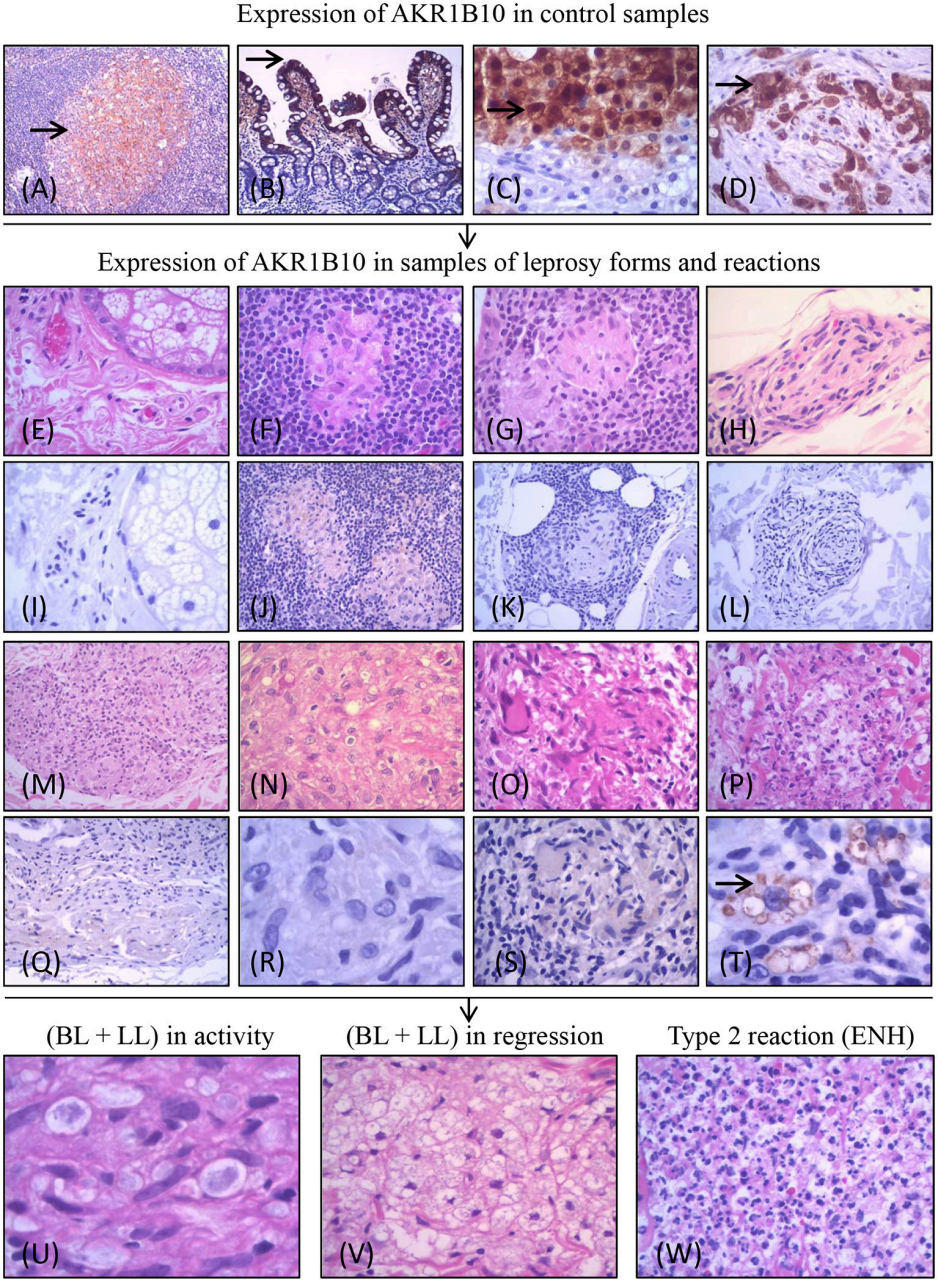
Experimental design of the project and expression of AKR1B10 (marked by →). Positive expression of AKR1B10 in controls: cells of the follicular center of the amygdala **(A)**, columnar cells in the duodenal mucosa **(B)**, in well-differentiated cells of hepatocellular carcinoma **(C)**, and in the carcinoma of pancreatic ducts **(D)**. Negative expression (absence or weak expression) in samples of healthy controls (HC) **(E,I)** and in different forms of leprosy [TT **(F,J)**; BT **(G,K)**; BB **(H,L)**; BL **(M,Q)**; LL **(N,R)**] and in R1 **(O,S)**. Positive expression of AKR1B10 (moderate or strong expression) in intracytoplasmic vacuoles of macrophages in R2 **(P,T)**. Expression in samples of lepromatous side (BL and LL) lesions in activity (L-side) (*n* = 46; **U**), lepromatous side lesions (BL and LL) in regression (L side T) (*n* = 45; **V**), and R2 lesions (*n* = 115; **W**).

The experimental design is shown in Figure 1. IHC was performed as described using the EnVision indirect method kit (Dako, California, USA) following the manufacturer's recommendations. Monoclonal anti-AKR1B10 was used (EPR14421 clone, 1:500, Abcam, Cambridge, UK) to measure the expression of AKR1B10 in all samples. Initially, as an external positive control, the antibody was tested in samples of the amygdala, duodenal mucosa, well-differentiated hepatocellular carcinoma, and pancreatic ductal adenocarcinoma (Figures 1A–D).

After verifying the activity of anti-AKR1B10 in these controls, we evaluated the expression of AKR1B10 in the same samples of leprosy lesions and healthy skin as in our previous study. We had observed upregulation of AKR1B10 mRNA in R2 samples compared with biopsies of other leprosy types along the R&J spectrum [HC (9 samples); TT, BT, BB, and BL (10 samples each); LL (4 samples)], R1 biopsies (14 samples), and R2 biopsies (10 samples) [(23); Figures 1E–T]. In a subsequent analysis, we verified our observations in skin biopsies from patients with lesions on the lepromatous side of the R&J spectrum (BL and LL) before treatment (L side; *n* = 46) and after initiation of treatment (L side-T) (*n* = 45) and those with type 2 reactional lesions (*n* = 115; Figures 1U–W).

**Figure 2:**
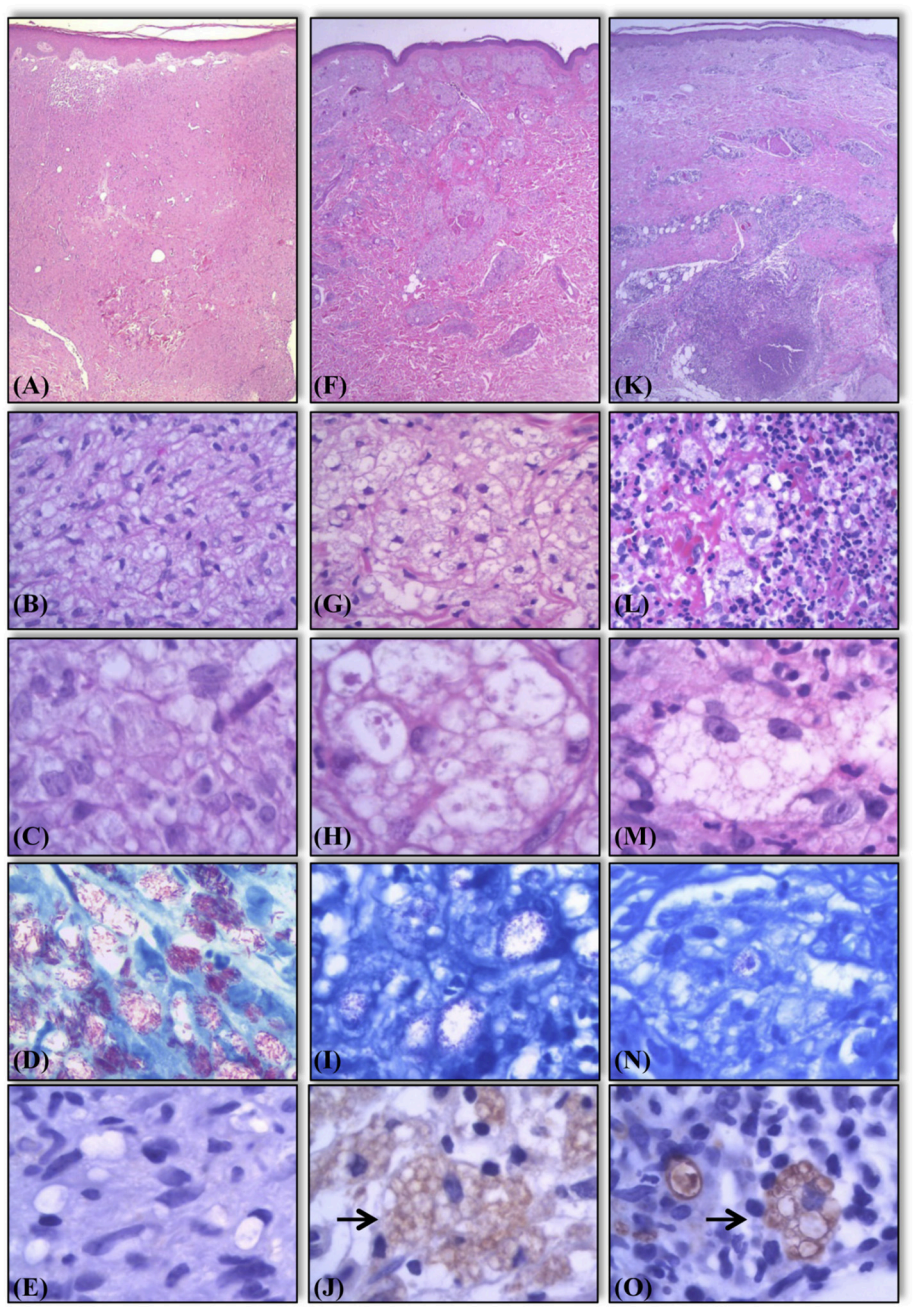
Histological and bacilloscopic characteristics of lesions of lepromatous side in activity, lepromatous side in regression, and R2 lesions. Active lesions with vacuolated macrophages containing a large number of solid bacilli **(A–D)**. Lesions in regression showing multivacuoled macrophages containing a large number of multifragmented bacilli **(F–I)**. R2 lesions, the macrophages display characteristics similar to those seen in lesions in regression undergoing an influx of neutrophils **(K–N)**. Expression of AKR1B10 in the same samples. Negative expression in samples of lepromatous side in activity **(E)**. Positive expression (marked by →) in samples of lepromatous side lesions in regression **(J)**, and R2 lesions **(O)**. Hematoxylin-eosin staining **(A–C, F–H, K–M)**. Fite-Faraco staining for bacilloscopy **(D,I,N)**.

All samples were obtained from the Lauro de Souza Lima Institute pathology laboratory archives of leprosy cases that were diagnosed from 2005 to 2016. The L side samples were obtained from patients with borderline lepromatous (BL) or lepromatous (LL) lesions, as confirmed by Fite-Faraco staining and showing well-stained solid bacilli before undergoing treatment. The L side T samples belonged to patients with lepromatous lesions (BL and LL) in the initial phase of their multidrug therapy or up to 2 years after the end of treatment. The R2 samples were also taken from patients with lesions on the lepromatous side (BL and LL) of the R&J spectrum after they had begun treatment but with the characteristic histological features that are associated with the type 2 reaction (Figure 2). Sequential sections of all samples were subjected to hematoxylin-eosin (HE) and Fite-Faraco staining (for bacilloscopy) and then IHC using anti-AKR1B10. All cases were reviewed to confirm their bacilloscopic index, their R&J classification, the presence of R2, and regression status (19, 21, 22). Samples from patients who had undergone 5 or more years of treatment were not included.

This study adhered to the Declaration of Helsinki and was approved by the Research Ethics Committees of Lauro de Souza Lima Institute, certified by CAAE 79664917.9.0000.5475. The clinical data of the patients are detailed in Supplement 1.

**Figure 3:**
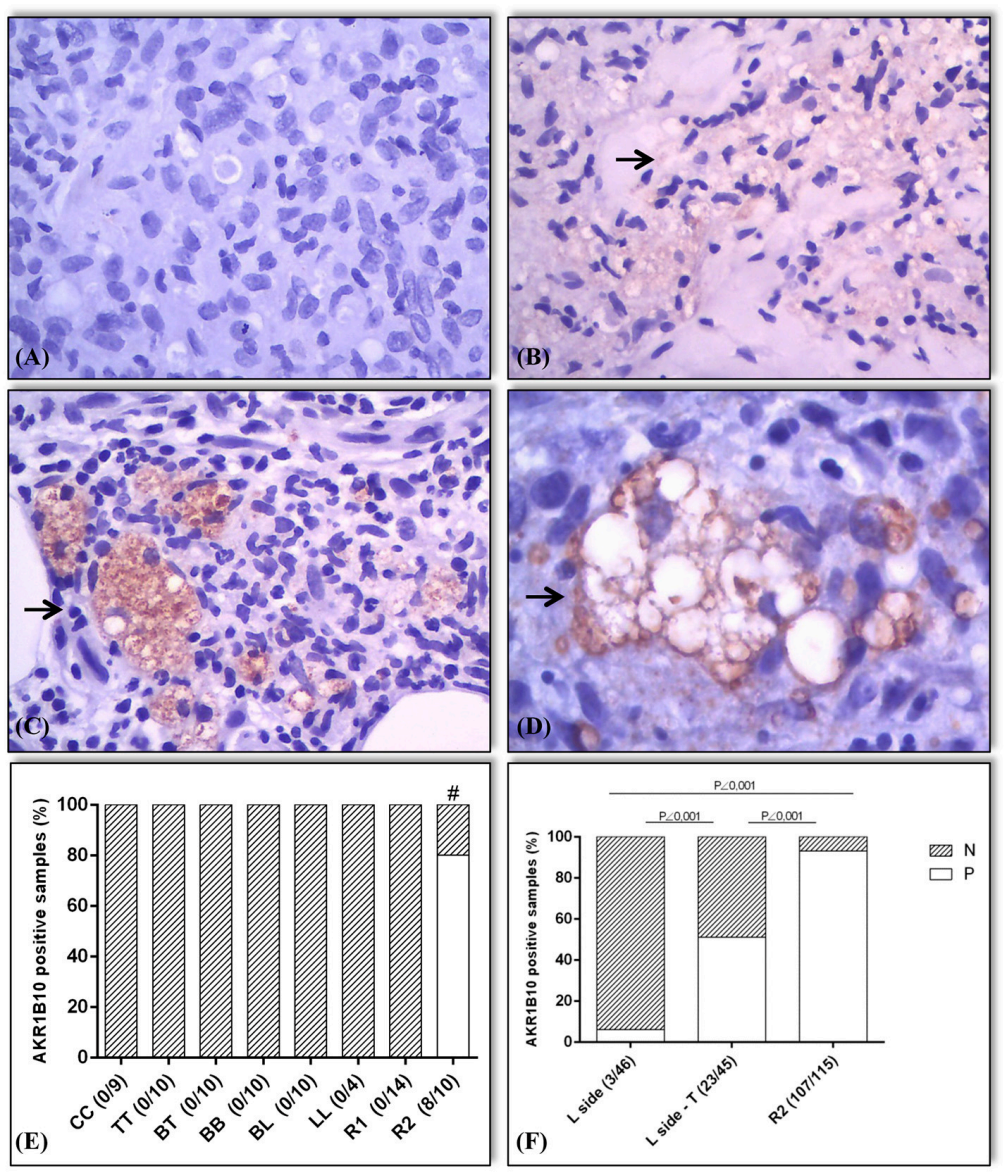
Expression pattern (marked by →) and expression values of AKR1B10 in positive samples. The samples were classified as negative for AKR1B10 when its expression was absent (0) or weak (1+; **A,B**). Positive expression of AKR1B10 (2+ or 3+ on the scale; **C,D**). In the preliminary assessment **(E)**, immunostaining was negative in all samples of HC, TT, BT, BB, BL, LL, and R1. It was positive in 8 out of 10 samples of R2 (80%), with a significant difference between groups (^#^ R2 vs. HC, R2 vs. TT, R2 vs. BT, R2 vs. BB, and R2 vs. BL, all with *p* = 0.0007; R2 vs. LL, *p* = 0.015; R1 vs. R2, *p* < 0.0001). In later evaluation **(F)**, there was positive expression in 3 out of 46 active lepromatous (BL and LL) samples (6%) (L side), 23 out of 45 samples of BL + LL in regression after treatment (L side-T) (51%), and 107 out of 115 samples of R2 (93%), with a significant difference between the L side samples and L side-T (*p* < 0.0001), R2 and L side (*p* < 0.0001), and between R2 and L side-T groups (*p* < 0.0001).

When the skin samples or the inflammatory cells in granulomas showed a weak/unclear signal (rated 1+ on a scale of 0 to 3+) or a lack of staining for AKR1B10, they were considered negative (Figures 3A,B). Moderate or strong (2+/3+) staining for AKR1B10 was considered positive in these samples (Figures 3C,D). The sample slides were first scanned at low magnification (10×) and then at high magnification (40×) to identify the immunostained cells (Figures 3A–D). The data on the differential expression of AKR1B10 in each sample were evaluated by two observers. The expression was examined in the following tissues: epidermis, cutaneous adnexal glands, interstitium, blood vessel walls, endothelium, subcutaneous adipose tissue, and cells in the inflammatory infiltrates that constituted the leprosy granulomas. Comparisons between groups were made using Fisher's exact test (GraphPad Prism 6.05; GraphPad Software, Inc., San Diego, California, USA; Figures 3E,F).

## Results

**Figure 4:**
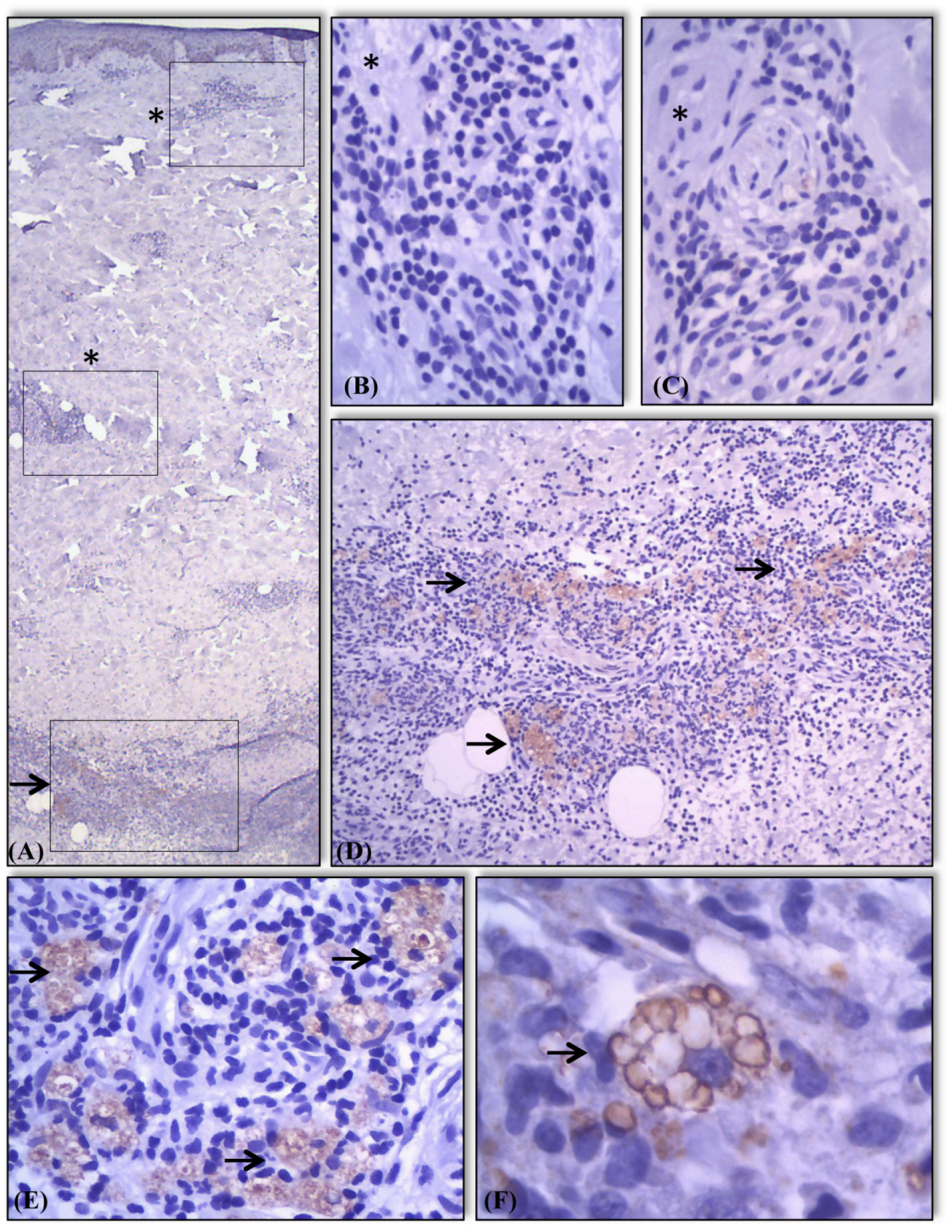
Expression of AKR1B10 in different components of skin and granulomas in the samples. Absence (marked with a^*^) of AKR1B10 expression in most components of skin and in some granulomas **(A–C)**. Positive expression (marked with a →) in granulomas present in other areas of the same sample **(A,D)**. Moderate or strong expression of marker almost exclusively in the cytoplasm of macrophages present in lesions of lepromatous lesions in regression **(E)** and R2 **(F)** showing staining of intracytoplasmatic vacuoles/lysosomes **(F)**.

The data from this study are summarized in Figures 1–4. The control samples showed moderate (2+) to strong (3+) immunostaining of AKR1B10 (Figures 1A–D). Of the leprosy lesions and healthy skin samples from our previous study, which reported transcriptional upregulation of *AKR1B10* in R2 samples, all healthy controls (n = 9), leprosy lesion samples from the R&J spectrum (10 TT, BT, BB, and BL samples; 4 LL samples), and R1 samples [*n* = 14; Figures 1E–S; (23)] were negative. However, AKR1B10 immunostaining was positive in 8 of 10 (8/10) R2 samples, corresponding to 80% positivity (Figure 1T). By pairwise comparison between several groups, there was a significant difference in AKR1B10 positivity (R2 vs. HC, R2 vs. TT, R2 vs. BT, R2 vs. BB, and R2 vs. BL, *p* = 0.0007; R2 vs. LL, *p* = 0.015; and R1 vs. R2, *p* < 0.0001; Figures 3E,F).

Among L side lesions, 3 of 46 (3/46) samples were positive by immunostaining, corresponding to 6% of all samples. L side T lesions were positive in 23 of 45 (23/45) samples (51%). Similarly, among R2 lesions, 107 of 115 (+107/115) samples showed positivity (93%) (Figures 2, 3F). Moreover, there was a significant difference in positivity between L side and L side T groups (*p* < 0.0001), R2 and L side groups (*p* < 0.0001), and R2 and L side T groups (*p* < 0.0001; Figure 3F).

Next, we examined the expression of AKR1B10 in various tissues and inflammatory infiltrates of leprosy but failed to detect it in any component of the skin or subcutaneous tissue. AKR1B10 was observed nearly exclusively in the cytoplasm of macrophages. Some macrophages showed intense expression of AKR1B10 in their intracytoplasmic vesicles, such as lysosomes and phagosomes (Figures 1S–T, 2J–O, 3C,D, 4F). In several samples, AKR1B10 was expressed in the neural branches that were involved in leprosy-associated inflammatory processes. In these cases, morphologically, AKR1B10 was predominantly expressed in the macrophages that permeated the neural branches that had been infected by parasites. Supplement 2 shows the data on the expression of AKR1B10 in these samples and their respective statistical values.

AKR1B10 expression was not homogeneous between clusters of inflammatory cells (Figure 4). In R2 granulomas, AKR1B10 was detected mostly in multivacuoled macrophages involving a central area of microabcesses and some were detected within microabscesses (Figures 4D,E). AKR1B10 was absent from lymphocytes, plasmocytes, neutrophils, and other cells that constituted the leprosy granulomas (Figure 4).

## Discussion

*AKR1B10* regulates several metabolic processes, based on its function in the metabolism of lipids and aldehydes (1, 2). Its dysregulation is associated with the development of various cancers. The function of *AKR1B10* in the development of inflammatory and infectious diseases is unknown (4, 9, 14, 15).

The mechanism of AKR1B10 varies between neoplasias (11). In pancreatic ductal neoplasms, AKR1B10 is overexpressed in precursor lesions and invasive carcinoma. Further, silencing of AKR1B10 is associated with greater apoptosis and decreased activation of *KRAS* and many of its effectors (7). In hepatocellular carcinoma, the expression of AKR1B10 is regulated through the activation of β-catenin signaling (24). In breast carcinoma, AKR1B10 promotes the migration and invasion of neoplastic cells by stimulating ERK signaling (25, 26). Inhibition of AKR1B10 constrains the growth of pancreatic cancer through modulation of the KRAS-E-cadherin pathway (27). Moreover, AKR1B10 participates in the activation of several mechanisms of drug resistance during the treatment of various cancers (5, 7, 28, 29).

There are few reports on the participation of AKR1B10 in the development of non-neoplastic diseases. The significantly higher expression of AKR1B10 in peripheral blood mononuclear cells (PBMCs) in diabetic nephropathy patients vs. healthy controls suggests that AKR1B10 mediates the development and progression of diabetic nephropathy (14). With regard to infectious diseases, AKR1B10 is overexpressed in hepatitis B and C and is linked to the development of hepatocellular carcinoma (9, 30–32). In inflammatory bowel disease, the loss of AKR1B10 correlates with chronic colitis and the development of colitis-related carcinoma (4).

There is little information on the function of AKR1B10 in leprosy. We recently published a study on the expression of genes in skin biopsy samples along the R&J spectrum of the disease and in its reactional forms (23). *AKR1B10* was upregulated only in R2 samples. Also, several miRNAs were differentially expressed in the spectral and reactional forms of leprosy. Based on our analysis of mi/mRNA expression, we noted that the expression patterns of hsa-miRNA-142-3p and AKR1B10 opposed each other (33). These results indicate that the expression of AKR1B10 is regulated by microRNA (33). The regulation of mRNA expression by miRNAs is complex, and there are also other mechanisms of epigenetic regulation of gene expression, necessitating further studies to determine whether there is an interaction between hsa-miR-142-3p and AKR1B10 in leprosy.

In macrophages, AKR1B10 expression was nearly confined to the cytoplasm, especially around the membranes of intracytoplasmic vacuoles (lysosomes; Figures 1–4). In normal tissues and neoplasms, AKR1B10 is expressed in various types of cells—primarily epithelial lineage cells—whereas in leprosy, it is expressed in macrophages. In macrophages, AKR1B10 is secreted through lysosomes through a non-classical pathway, increasing its level in the serum of patients with breast cancer (16). AKR1B10 localizes to the cytoplasm and translocates to lysosomes via heat shock protein 90 (HSP90), a chaperone molecule. After fusion of the lysosome to the plasma membrane, AKR1B10 is released into the interstitium and then into the bloodstream and thus can be measured in serum (17)

There is a little knowledge about the pathophysiological mechanisms that trigger an R2 reaction (19, 21). R2 occurs in lepromatous lesions (BL and LL), usually after treatment is initiated. It is believed that during bacillary fragmentation, which can be spontaneous or caused by multidrug therapy, antigens that stimulate the production of antibodies are released into the blood and interstitial fluids (19, 21). Due to unknown mechanisms of the abrupt release of antigens from macrophages, an antigen-antibody reaction occurs in the interstitium, accompanied by complement fixation and stimulation, developing into an acute inflammatory reaction with varying intensities (19, 21).

The histological features of R2 reflect an acute or subacute inflammatory reaction in the foci of lepromatous granulomas during regression (19). In general, R2 is a well-defined example of an acute inflammatory reaction, comprising vascular dilation, endothelial swelling, and serofibrinous and neutrophilic exudation, disrupting pre-existing granulomas. The more intense processes include the development of thrombi in the venocapillary territory, vasculitis, and the formation of microabscesses (19). Like most antigens in the extravascular space, the antigen-antibody reaction and complement fixation occur in parasitized tissues, triggering an acute inflammatory reaction in which neutrophils undergo chemotaxis and subsequently migrate to the interstitium to induce phagocytosis and destruction of the immune complexes (19).

The function of AKR1B10 in leprosy is unknown. It is unclear whether its expression in macrophages is associated with the development of R2. Nonetheless, our results indicate that AKR1B10 predominates in macrophages on the start of treatment (L-side T) and in R2 lesions. It is possible that the expression of AKR1B10 in the lysosomes of macrophages is phenomenon precipitating event, resulting from the intense fragmentation of bacilli by chemotherapy, and triggers an R2 reaction. Our data show that the macrophages in leprosy lesions that are in regression after treatment (showing no evidence of R2) express AKR1B10, which is nearly absent in active macrophages in the lesion (Figure 3).

There are significant histological differences between active lesions and lesions that are in regression. In active lepromatous BL and LL lesions, macrophages have an M2 immunoprofile, with an eosinophilic, vacuolated cytoplasm that contains many solid, well-stained bacilli and fewer fragmented bacilli in the vacuoles and cytoplasm (Figures 2A–E). At the outset of treatment, there is intense and progressive multivacuolation of the cytoplasm in macrophages. These vacuoles have distinct morphological features and a lysosomal profile by IHC, containing many multifragmented bacilli with varying intensities of Fite-Faraco staining. It is unknown how bacilli interfere with the expression of AKR1B10 in parasitized macrophages. However, our findings indicate that the predominance of bacilli in active lesions blocks AKR1B10 expression in the cytoplasm and the formation of vacuoles in macrophages in these active lesions. When treatment begins, the fragmentation of bacilli disrupts this balance; consequently, the macrophages express AKR1B10.

The expression of AKR1B10 could lead to the exposure of antigens on the surface of macrophages or enhance the fusion of intracytoplasmic vacuoles that contain a large number of fragmented bacilli in the plasma membrane and then release them into the interstitium, or it might promote the disintegration of macrophages. If AKR1B10 functions in the development of R2, substances that inhibit it could act on parasitized macrophages that retain fragmented bacilli in the lysosomes, thus retarding bacterial clearance and blocking the factors that trigger the onset of reactional episodes or decreasing the frequency and intensity of R2. Thus, even if R2 develops, the reaction episodes would be less intense, allowing better management of reactions and mitigation of clinical sequelae, which are caused primarily by intense reaction episodes that destroy neural branches and tissues.

Leprosy reactions are significant in the development of leprosy (19, 20). There is no marker, serological or *in situ*, that identifies or predicts the occurrence of reactional episodes (20). The most effective drug that is used to treat R2 is thalidomide (34). Because it is teratogenic, its use is restricted, and it is sometimes replaced with less effective drugs, such as corticosteroids, which have major side effects [immunosuppression, diabetes, obesity, osteoporosis, glaucoma, etc.; (35)].

Because AKR1B10 has been identified in the blood as a cancer marker, it is possible that it has a similar function in R2 (13). Whether anti-AKR1B10 drugs block the activation of R2 or eventually reduce the intensity and frequency of reactional episodes should be examined. There are several reports on various types of drugs, from herbal drugs to monoclonal antibodies, with anti-AKR1B10 activity (27, 30, 32, 36–50).

In summary, *AKR1B10* is expressed in the skin lesions of patients with leprosy, predominantly in lepromatous lesions that are in regression, especially those that are associated with R2. Further studies should determine whether AKR1B10 is important for triggering the R2 reaction and whether it can be used as a marker or therapeutic target for the prevention and clinical management of R2 episodes of leprosy.

## Author contributions

CS and AB conceived the project. CG and CS performed clinical evaluation of patients and performed biopsy procedures. CS performed histopathological and immunohistochemical analyses. CS, AB, AT, LF, and PR were responsible for data analysis. CS and PR wrote the manuscript. All authors reviewed the paper and provided their inputs.

### Conflict of interest statement

The authors declare that the research was conducted in the absence of any commercial or financial relationships that could be construed as a potential conflict of interest.
