# Supplementary material for: Potential of AKR1B10 as a Biomarker and Therapeutic Target in Type 2 Leprosy Reaction
Source: Front Med (Lausanne). 2018 Sep 24;5:263. doi: 10.3389/fmed.2018.00263 (PMC6166685; doi:10.3389/fmed.2018.00263)
Supplement: Supplementary file 2 [file Data_Sheet_2.PDF]

Supplement 2

2.1- First analysis. Expression of AKR1B10 in granuloma cells or skin tissues. Skin samples (biopsies per punch) of patients with leprosy forms (TT, BT, BB, BL and LL), reactions (R1 and R2) and control samples from healthy patients (HC) (n = 67). Immunostaining was negative (0 or 1+) in all HC, TT, BT, BB, BL, LL and R1 samples. Eight of 10 samples of R2 (80%) were positive (2+ or 3+), with a significant difference when compared to the other groups (R2 x HC, R2 x TT, R2 x BT, R2 x BB and R2 x BL: p = 0.0007; R2 X LL: p = 0.015 and R2 x R1: p <0.0001).

Marker	HC (n=9)	TT (n=10)	BT (n=10)	BB (n=10)	BL (n=10)	LL (n=4)	R1 (n=14)	R2 (n=10)
**AKR1B10**	Granuloma (not)<br>Nerve: (-)<br>endoneurium ( )<br>perineurium( )<br>Blood vessel: (-)<br>endothelium ( )<br>wall ( )<br>Interstitium: (-)<br>Pilosebaceous units: (-)<br>Epidermis: (-)<br>Glands:(-)	Granuloma (+)<br>Nerve: (-)<br>endoneurium ( )<br>perineurium( )<br>Blood vessel: (-)<br>endothelium ( )<br>wall ( )<br>Interstitium: (-)<br>Pilosebaceous units: (-)<br>Epidermis: (-)<br>Glands:(-)	Granuloma (+)<br>Nerve: (-)<br>endoneurium ( )<br>perineurium( )<br>Blood vessel: (-)<br>endothelium ( )<br>wall ( )<br>Interstitium: (-)<br>Pilosebaceous units: (-)<br>Epidermis: (-)<br>Glands:(-)	Granuloma (+)<br>Nerve: (-)<br>endoneurium ( )<br>perineurium( )<br>Blood vessel: (-)<br>endothelium ( )<br>wall ( )<br>Interstitium: (-)<br>Pilosebaceous units: (-)<br>Epidermis: (-)<br>Glands:(-)	Granuloma (+)<br>Nerve: (+)<br>endoneurium ( )<br>perineurium( )<br>Blood vessel: (+)<br>endothelium ( )<br>wall ( )<br>Interstitium: (-)<br>Pilosebaceous units: (-)<br>Epidermis: (-)<br>Glands:(-)	Granuloma (+)<br>Nerve: (-)<br>endoneurium ( )<br>perineurium( )<br>Blood vessel: (-)<br>endothelium ( )<br>wall ( )<br>Interstitium: (-)<br>Pilosebaceous units: (-)<br>Epidermis: (-)<br>Glands:(-)	Granuloma (+)<br>Nerve: (-)<br>endoneurium ( )<br>perineurium( )<br>Blood vessel: (-)<br>endothelium ( )<br>wall ( )<br>Interstitium: (-)<br>Pilosebaceous units: (-)<br>Epidermis: (-)<br>Glands:(-)	Granuloma (+)<br>Nerve: (-)<br>endoneurium ( )<br>perineurium( )<br>Blood vessel: (-)<br>endothelium ( )<br>wall ( )<br>Interstitium: (-)<br>Pilosebaceous units: (-)<br>Epidermis: (-)<br>Glands:(-)

1-HC (n = 9). Intensity of AKR1B10 expression in the samples. Negative (0 and 1+). Positive (2+ and 3+): (0/9); (1 + / 0); (2 + / 0) and (3 + / 0).

Granuloma (absence of granulomas) (not)

Nerve: (-)

endoneurium ( )

perineurium( )

Blood vessel: (-)

endothelium ( )

wall ( )

Interstitium: (-)

Pilosebaceous units: (-)

Epidermis: (-)

Glands:(-)

Summary HC: absence of AKR1B10 expression in all skin components.

2-TT (n = 10). Intensity of AKR1B10 expression in the samples. Negative (0 and 1+). Positive (2+ and 3+): (0/5); (1/5); (2/0) e (3/0).

Granuloma (0 or 1+) *

Nerve: (-)

endoneurium ( )

perineurium( )

Blood vessel: (-)

endothelium ( )

wall ( )

Interstitium: (-)

Pilosebaceous units: (-)

Epidermis: (-)

Glands:(-)

Summary TT(*): Weak expression only in macrophages. Absence of AKR1B10 expression in all skin components.

3-BT (n = 10). Intensity of AKR1B10 expression in the samples. Negative (0 and 1+). Positive (2+ and 3+): (0/8); (1/2); (2/0) e (3/0).

Granuloma (0 or 1+) **

Nerve: (-)

endoneurium ( )

perineurium( )

Blood vessel: (-)

endothelium ( )

wall ( )

Interstitium: (-)

Pilosebaceous units: (-)

Epidermis: (-)

Glands:(-)

Summary BT (**): Weak expression only in macrophages. Absence of AKR1B10 expression in all skin components.

4-BB (n = 10). Intensity of AKR1B10 expression in the samples. Negative (0 and 1+). Positive (2+ and 3+): (0/9); (1/1); (2/0) e (3/0).

Granuloma (0 or 1+) ***

Nerve: (+)

endoneurium (+)

perineurium( )

Blood vessel: (-)

endothelium ( )

wall ( )

Interstitium: (-)

Pilosebaceous units: (-)

Epidermis: (-)

Glands:(-)

Summary BB (***): Weak expression in macrophages and neural branches. Absence of AKR1B10 expression in all skin components.

5-BL (n = 10). Intensity of AKR1B10 expression in the samples. Negative (0 and 1+). Positive (2+ and 3+): (0/9); (1/1); (2/0) e (3/0).

Granuloma (0 or 1+) ****

Nerve: (+)

endoneurium (+)

perineurium( )

Blood vessel: (-)

endothelium ( )

wall ( )

Interstitium: (-)

Pilosebaceous units: (-)

Epidermis: (-)

Glands:(-)

Summary BB (****): Weak expression in macrophages and neural branches. Absence of AKR1B10 expression in all skin components.

6-LL (n = 4). Intensity of AKR1B10 expression in the samples. Negative (0 and 1+). Positive (2+ and 3+): (0/3); (1/1); (2/0) e (3/0).

Granuloma (0 or 1+) *****

Nerve: (-)

endoneurium ( )

perineurium( )

Blood vessel: (-)

endothelium ( )

wall ( )

Interstitium: (-)

Pilosebaceous units: (-)

Epidermis: (-)

Glands:(-)

Summary BT (*****): Weak expression only in macrophages. Absence of AKR1B10 expression in all skin components.

7-R1 (n = 14). Intensity of AKR1B10 expression in the samples. Negative (0 and 1+). Positive (2+ and 3+): (0/4); (1/10); (2/0) e (3/0).

Granuloma (0 or 1+) ******

Nerve: (-)

endoneurium ( )

perineurium( )

Blood vessel: (-)

endothelium ( )

wall ( )

Interstitium: (-)

Pilosebaceous units: (-)

Epidermis: (-)

Glands:(-)

Summary BT (******): Weak expression only in macrophages. Absence of AKR1B10 expression in all skin components.

8-R2 (n = 10). Intensity of akr1b10 expression in the samples. Negative (0 and 1+). Positive (2+ and 3+): (0/0); (1/2); (5/5) e (3/3).

Granuloma (1+ to 3+) *******

Nerve: (1+) *******

endoneurium (+)

perineurium( )

Blood vessel: (-)

endothelium ( )

wall ( )

Interstitium: (-)

Pilosebaceous units: (-)

Epidermis: (-)

Glands:(-)

Summary R2 (*******): Weak expression in neural branches. Moderate(2+) or intense(3+) expression in macrophages. There was no expression of the marker on the other skin components and neutrophil agglomerates (microabscesses).

HC (n=9)	0/9
TT (n=10)	0/5 and 1+/5
BT (n=10)	0/8 and 1+/2
BB (n=10)	0/9 and 1+/1
BL (n=10)	0/9 and 1+/1
LL (n=4)	0/9 and 1+/1
R1 (n=14)	0/4 and 1+/10
R2 (n=10)	1+/2 and 2 + or 3+/8

2.2- Second analysis (late analysis). Expression of AKR1B10 in granuloma cells or skin tissues. Skin samples (biopsies per punch) of patients with classified as virchowian active leprosy (L side), lepromatous leprosy strain in post-treatment regression (L side T) and reaction type 2 (R2) (n = 206). There was positive expression in 3 out of 46 active BL+LL (L side) samples (6%), 23 out of 45 samples of BL+LL in regression after treatment (L side-T) (51%), and 107 out of 115 samples of R2 (93%), with a significant difference between the L side and L side-T ($p < 0.0001$), R2 and L side ($p < 0.0001$), and between R2 and L side-T groups ($p < 0.0001$).

Marker	L side (n=46)	L side T (n=45)	R2 (n=115)
AKR1B10	Granuloma (+) 3/46 (6%)<br><br>Nerve: (-)<br>endoneurium ( )<br>perineuro( )<br><br>Blood vessel: (-)<br>endothelium ( )<br>wall ( )<br><br>Interstitium: (-)<br><br>Pilosebaceous units: (-)<br><br>Epidermis: (-)<br><br>Glands:(-)	Granuloma (+) 23/45 (51%)<br><br>Nerve: (-)<br>endoneurium ( )<br>perineuro( )<br><br>Blood vessel: (-)<br>endothelium ( )<br>wall ( )<br><br>Interstitium: (-)<br><br>Pilosebaceous units: (-)<br><br>Epidermis: (-)<br><br>Glands:(-)	Granuloma (+) 107/115 (93%)<br><br>Nerve: (-)<br>endoneurium ( )<br>perineuro( )<br><br>Blood vessel: (-)<br>endothelium ( )<br>wall ( )<br><br>Interstitium: (-)<br><br>Pilosebaceous units: (-)<br><br>Epidermis: (-)<br><br>Glands:(-)
